# Accessibility to sexual and reproductive health and rights education among marginalized youth in selected districts of Tanzania

**DOI:** 10.11604/pamj.supp.2016.25.2.10922

**Published:** 2016-11-26

**Authors:** David Paul Ngilangwa, Sharanya Rajesh, Mercy Kawala, Rita Mbeba, Benatus Sambili, Serafina Mkuwa, Rita Noronha, Alfred Jackson Meremo, Josephat Nyagero

**Affiliations:** 1 Amref Health Africa in Tanzania; 2 School of Medicine, College of Health Sciences, University of Dodoma, Dodoma, Tanzania; 3 Amref Health Africa, Headquarters, Nairobi, Kenya

**Keywords:** Marginalized youths, access, SRHR, school dropout, Tanzania

## Abstract

**Introduction:**

Access to information, education and services is central in the promotion of sexual and reproductive health and rights (SRHR) among young people. It enables young people make informed choices on sexuality matters, hence reduce teenage pregnancies and sexually transmitted infections (STIs). This study assessed accessibility of SRH rights’ information among marginalized young people in three municipalities of Iringa, Ilala and Kinondoni in Tanzania.

**Methods:**

A cross-sectional study design using mixed methods was conducted in 2013 in three selected districts of Tanzania. We randomly selected 398 young people including those with disabilities to partake in quantitative survey while 48 community members were purposely selected for qualitative part. Quantitative data analysis was done using descriptive statistics and chi square test with the assistance of the Statistical Package for Social Science(SPSS) version 20, while qualitative data was thematically analyzed.

**Results:**

There were 396 (99%) participants (144 Males and 251 females), with a mean age of 20.1years. The majority were out of school female, cohabiting and had completed secondary education. Overall, 317 (79.6%) had SRH rights knowledge. The leading sources of SRH rights information were peer educators (36.7%) and radio (22.8%). Awareness regarding laws and policies related to SRH right was 55.1%. However, young people living with HIV and school truants had more access to SRHR education and services than the other youth groups(χ2 30.69, p< 0.0001). The qualitative study revealed that parents and teachers perceived themselves to be incompetent in providing SRH information to their youngsters despite of being mostly trusted.

**Conclusion:**

Access to SRH rights information is high but decreases when it is disaggregated across different age groups. There is a need for diversified approach for reaching specific groups of young people depending on their needs and circumstances

## Introduction

In Sub-Saharan Africa (SSA), young persons aged 10 to 24 accounts for 30 percent of the population [1]. Furthermore, Africa is set to add 1.3 billion people to the world by 2050 and nearly all of the growth is expected to be concentrated in the poorest regions of SSA [1]. Given their proportion, energy and resourcefulness, young people’s health and, sexual and reproductive health in particular, has a significant impact on the long-term national development agenda of a country. Access to information, education and services is central in the promotion of sexual and reproductive health and rights (SRHR) for young people. Globally, and in SSA region in particular, many young people lack education and have poor access to services related to SRHR [2].

Poor access to SRHR has been associated with young people’s vulnerability to sexual health risks, such as early pregnancies and sexually transmitted diseases [3]. Adolescent birth rates seem to be the highest where the youth population is the highest [1]. According to the Africa Youth Report, in 2011 about 28 percent of women aged 20-24 in the SSA region gave birth before the age of 18, with almost half of them having had their first sexual experience before their 15th birthday [4]. Early pregnancy is partly attributable to low use of modern contraceptive methods. According to a report by the United Nations Population Fund, in 2014, more than four in 10 women of reproductive age (15-49) in SSA wanted to avoid pregnancy [5]. However, over half of these women-55 million-were not using an effective contraceptive method [5].

In East Africa, Tanzania has the second youngest population with a median age of the population being 18 years [6]. The 10-24 year old youth population constitutes 32 percent of the total population [1]. Globally, fertility rates have been dropping since the 1950s, from an average of six children per woman to about 2.5 today [1]. However, the fertility rate in Tanzania, as elsewhere in the SSA region, is generally high with an average of 5.2 births per women [7]. There is also a high adolescent fertility rate with 81 births per thousand women aged 15-19 [6]. Furthermore, just half of pregnant young women give birth in a health facility with the assistance of health professionals [8]. The proportion of sexually active young people with multiple sexual partners is also relatively high, with 17.4 percent of males aged 15-24 reporting having had two or more partners in the past 12 months, and less than half of them used a condom during their last sexual encounter [8]. According to a 2014 report by Tanzania Commission for AIDS, the Human immunodeficiency virus (HIV) prevalence rate in Tanzania was 5.3 percent with women bearing the brunt of the epidemic at 6.3 percent as compared to 3.9 percent among men [9].

A targeted focus on adolescent sexual and reproductive health has born positive results in recent years. Data from the Tanzania Demographic Health Survey (TDHS) 2010, for example, showed that the adolescent pregnancy rates has declined by 12 percent, from 132 per 1000 females aged 15-19 years in 2004 to 116 per 1000 females in 2010 [8]. Nevertheless, Tanzania still has one of the highest adolescent pregnancy and birth rates in the world. This is partly attributable to early marriages with an average of almost two out of five girls being married before their 18th birthday [6]. About 37% of women aged 20-24 were married or were in union before the age of 18 [6]. This implies that the sexual and reproductive health needs of some young people are not being met adequately, particularly for young adolescents below the age of 18.

Though information, education and services related to sexual and reproductive health are limited to young people in general, they are further limited to certain marginalized groups such as young people living with HIV/AIDS, orphans, young people in rural areas, and people with disability. Evidence of SRHR status of marginalized youth in Africa and especially in Tanzania is lacking. Adolescents with disability are found to be more vulnerable to sexual abuse, unplanned pregnancy, HIV and sexually transmitted diseases [10–12]. Homeless adolescents are found to face a higher risk of HIV infection [13, 14]. In SSA, adolescent girls belonging to financially backward families have a higher rate of early pregnancy than girls belonging to families with wealth [15].

Amref Health Africa had been implementing a 3-years the rights based project on SRHR called TUITETEE project in three municipalities; two in Dar es Salaam (Kinondoni and Ilala Municipal councils) and one in Iringa Region (Iringa Municipal Council) in Tanzania. Its focus is to support reproductive health and rights of young people aged 10-24 years including making pregnancy safer for women. The project targeted approximately 100,000 people including 100 youths with disabilities and those living with HIV. This study therefore examines the gaps in access to SRHR communication among marginalized youth in selected districts of Tanzania who were a part of the TUITETEE project. Findings obtained from this study aimed at informing ongoing programs on SRHR as well as generating new scientific insights on the best approaches to reach the marginalized young people with information and education on SRHR.

## Methods

### Study design, settings and sites

A cross-sectional descriptive survey was employed using quantitative and qualitative research methods. This study was conducted across three municipalities of Iringa, Kinondoni and Ilala districts of Tanzania with the study population identified within school settings, home settings and laborers at local businesses.

### Study population

The population study included all marginalized young people aged 10-24 years who were participating in the TUITETEE project. In the context of this study, marginalized youth included all young people with disabilities (deaf and visually impaired), young people living with HIV and AIDS, school truants and casual laborers, including food vendors and house maids.

*Sampling and sample size*: The sample size for this study was calculated using the Yamane (1967) formula, n=N/1+N(e)2 Where, n=required sample size, N=population size, e=level of precision. The sample size for this study was approximated at 398, allowing for a sampling error of 0.05 (95% confidence level). To significantly minimize sampling error, the sample size was adjusted to 400 participants for the quantitative study. Participants for the questionnaire were randomly selected by ward with each being represented by one primary and one secondary school. A list of marginalized youth across various categories was obtained from the youth networks participating in the TUITETEE project.

### Data collection

The strategies used to identify respondents for the qualitative study varied according to different youth groups they belonged to. For example, housemaids were mainly selected using a snowball approach with shops located within their vicinity of residential areas used as first reference points. Youth with disabilities were identified through their disability centers. Those with visual impairment were assisted to complete the questionnaires by research assistants. There were also sign language interpreters for those who had hearing impairment. Peer educators were located both in schools and out of school settings and were identified through youth centers.

Interviews for qualitative data were conducted with 3 Focus Group Discussions (FGDs) for peer educators and 21 in-depth interviews (IDIs) for parents, teachers and community leaders at the ward level being held. The data was collected using pre-tested tools.

Data collection was done by six trained research assistants (two in each district) under close supervision of the study investigators for two-three weeks. A stakeholders’ meeting was conducted with policy makers and Programme Managers from the Ministry of Health and Social Welfare (MoHSW) prior to study start to raise awareness about the study.

### Data management and analysis

*Quantitative data*: The coded quantitative data were entered into computer and subsequently analyzed using the Statistical Package for Social Sciences (SPSS) version 20.0. Data was analyzed using both descriptively and inferential statistics. Descriptive statistics were used to gauge the proportion of young people for various variables in the study including socio-demographic information, source of information and levels of access to information among the others. Inferential statistics such as Chi-square tests were applied to assess the variation among young people and between male and female youth across outcomes of the study.

*Qualitative data*: Data from the FGDs and IDIs proceedings were recorded and transcribed verbatim. They were then subjected to content thematic analysis. Codes and themes were identified after several reads and reviews of the transcripts. The themes were described and demonstrated using participants’ quotes perspectives about various aspects of SRHR.

Data validity and reliability were ensured by the field supervisor and investigators who reviewed all the questionnaires, interviews and FDGs scripts on daily basis to rectify any inconsistencies and ensure that intended questions have been answered properly. Feedbacks emanating from review of interviews were conveyed to the research assistants for the triangulation and improvement of quality of data collected. In addition, study used a separate independent interpreter to re-translate interview recordings where possible to ensure validity of interpretations.

#### Ethical considerations

The study was reviewed and approved by the Tanzania Medical Research Coordinating Committee. Permission was also sought from relevant municipal authorities and all logistic arrangements for the study were made in close consultation with relevant authorities. Written informed consent was obtained from each participant before enrolment into the study and after the study procedures and objectives were explained to them by the research assistants. Verbal consent was obtained from participants who could not see, read or write and was recorded by the research assistants.

Permission was sought from parents/guardians and/or teachers for all participants below the age of 18 years. Individual participants were assured of their freedom to participate and to drop out of the study at any time without any consequence. Information gathered from participants was handled confidentially and the whole research process adhered to the principal of anonymity by redacting personal identifiable information of the participants on the questionnaires, interviews and FGD transcripts.

## Results

### Participants’ socio-demographic characteristics

A total of 396 (99%) participants aged between 11¬-26 years, with a mean age of 20.1 years (SD ± 3.28), completed the required questionnaire. Almost two thirds (63.5%) of the participants were female. The majority (69.8%) of young people participating in the study were out of school youths. More than three quarters of the participants were either married (11.4%) or living with a partner (66.3%). The majority of participants had either completed primary education (39.4%) or secondary education (41.5%). The participants’ demographic information has been summarized in Table 1.

**Table 1:** Socio-demographic characteristics of study participants

Demographic Variable	N	%
***Sex***		
Male	144	36.5
Female	251	63.5
***Status of young people***		
Food vendors	78	19.6
House maids	46	11.6
School truants	109	27.5
Youth Living with HIV	48	12.1
Youth with disabilities	116	29.2
***District***		
Kinondoni	111	28.0
Ilala	102	25.8
Iringa	183	46.2
***Studentship***		
In school	116	30.2
Out of school	268	69.8
***Marital Status***		
Married	43	11.4
Living with a partner	250	66.3
Single	67	17.8
Separated	14	3.7
Widow/widowed	3	0.8
***Education Level***		
Below primary school	34	8.9
Primary education	150	39.4
Secondary education	158	41.5
Post-secondary education	39	10.2
***Religious Affiliation***		
Protestants	67	17.4
Catholics	155	40.5
Moslems	119	31.1
Other	31	8.1
None	9	2.3

N= Number of participants, %= Percentage of participants

### Sources of information about SRHR among young people

**Figure 1:**
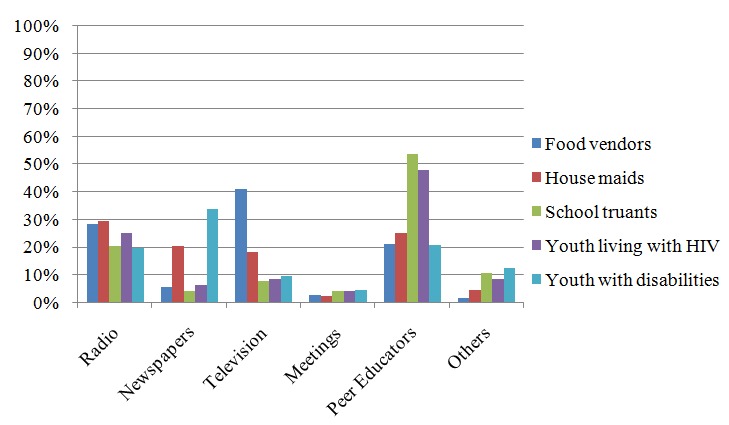
Young people's sources of information about SRHR-Individual sources versus Mass media outlets

Respondents were given several options regarding possible sources of information about sexual and reproductive health (SRH) and were requested to indicate the sources they have been receiving such information. The sources included parents/guardians, teachers, relatives, friends/peers, leaders and mass media outlets (newspapers, television, radio, etc.). Groups of people as sources of information were asked separately from media outlets. The results are summarized in Figure 1.

The leading source of information about SRHR was found to be peer educators with 36.7% of respondents accessing them. School truants were found to be accessing peer educators the most (53%), followed by young people living with HIV (48%). Radio was the second most popular mass media outlet for obtaining SRHR information (by 22.8% of the respondents). Different sources of media were used by different youth groups. The majority of young food vendors (41%) indicated television as their main source, compared to 30% of housemaids who indicated radio as their main media source of information and about a third of young people with disabilities indicated newspapers as their main source of SRHR information.

In the individual sources of SRHR information, it emerged that parents/guardians were the main source of information for the majority of young people, with the exception of youth with disabilities whose main source were teachers (indicated by 53% of the youth with disabilities). In both cases, individual sources and mass media outlets, the variation in the proportion of young people indicating them as sources of information about SRHR information was statistically significant χ^2^=379.35p<0.001for individual sources and χ^2^=378.30, p<0.001for media sources.

When respondents were asked to indicate whether they knew of any place where they could obtain information, education and/or services related to STDs and HIV/AIDS, 69.6% of them responded affirmatively. Less than a quarter (17.4%) of house maids knew any place where they could obtain information, education and/or information about STDs and HIV/AIDS. Chi square tests showed that the variation in the level of awareness of the place where information, education and services about STDs and HIV/AIDS was statistically significant (χ^2^=375.5, p< 0.001).

### Level of awareness about laws, policies and guidelines on SRHR

**Figure 2:**
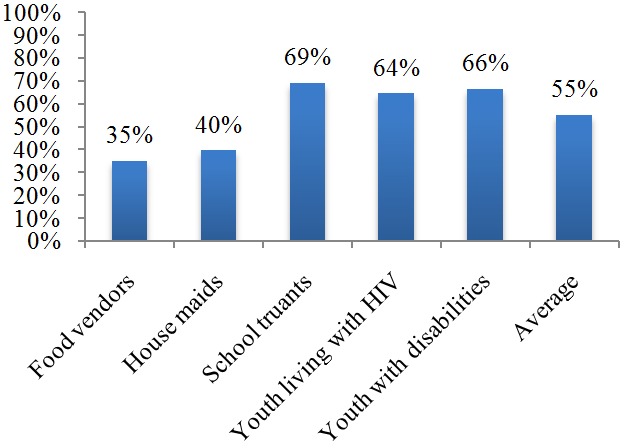
Percentage of young people who are aware of existing laws, policies and guidelines about SRHR

Respondents were also asked to indicate the extent to which they were aware of laws, policies and guidelines related to SRHR. On average, 55.1% of the respondents reported that they were aware of some laws, policies and guidelines related to SRHR. The level of awareness was high among school truants (69%), followed by youth with disabilities (66.4%) and youth living with HIV (64.4%). The level of awareness was low among food vendors (34.8%) and housemaids (39.5%). Chi square tests revealed that the variation in the level of awareness among the five youth groups was statistically significant (χ^2^=30.69, p <0.001). Figure 2

#### Perceived importance of SRHR information

The respondents observed that provision of appropriate information, education and services about SRHR would enable young people to avoid sexual health problems such as sexually transmitted infections, early pregnancies and HIV and AIDS. Parents in particular thought that many young people start sexual activities at young age because they did not receive proper education about SRH at home and in schools. Most of the examples about the risks associated with poor SRH information were discussed with respect to girls. Respondents’ quotes below are illustrative of their perceived importance of SRH information to young people.

*“…Sexual health information is important for young people because when they get it they know that I am now old enough, I can get pregnant, I can be infected with HIV. Without proper information about sexual health it is very easy for them to be trapped and eventually end up getting pregnant or HIV…”* (Parent, Iringa municipal).

*“This education (about SRH) is very, very important, there is no doubt about it. Many young people have lost their lives because of ignorance; they don’t know how to deal with this tricky world. These days a thirteen-year-old girl can be pregnant, if you ask why, it is because of lack of education about sexuality girls are unable to manage con men in the street”*. (Ward Executive Officer, Ilala municipal).

#### The extent and context of SRHR communication between parents/guardians and young people

Less than half (46.3%) of the respondents reported that they communicated with their parents/guardians on SRHR issues regularly, and 31.9% reported that they communicated rarely. The majority of young people (54%) indicated that they used face-to-face discussions as the major means of communication between them and their parents/guardians on matters related to SRHR. When the results were analyzed with respect to youth groups, it emerged that a higher proportion (62.5%) of youth living with HIV reported communicating with their parents/guardians on SRHR issues more regularly than other groups.

Overall, very little communication was reported between parents and young people regarding sexual health. This communication was mainly in form of warnings and directives from parents to their children. The content of this communication mainly centered on issues related to HIV and AIDS, sexually transmitted diseases and pregnancy.

*“We tell them to be aware of their days (menstruation), if they play around they will be in trouble.”* (Male parent, Iringa)

*“Parents tend to give different information to youngsters, depending where they may be. Those in Manzese stay very close to bars, for instance. The messages will have to be on preventing youngsters from being beer drinkers. Those in Mikocheni and Masaki like watching TV and other gadgets, they know a lot already, so what can a parent tell them?”* (Ward Leader, Ilala).

#### SRHR topics involved in communication between parents/guardians and young people

Respondents were provided with a list of eight most common topics on sexual and reproductive health, and they were asked to indicate the extent to which they discussed with their parents/guardians about them as indicated in Table 2. The least discussed topic was homosexuality with only 20.1% of the youth reporting that they discussed this topic with their parents/guardians frequently. The most discussed topic was HIV and AIDS with 46.5% of the respondents reporting having discussed this topic frequently. Housemaids reported discussing SRHR with their parents/guardians less frequently compared to other youth groups. A statistically significantly higher proportion of female youth (48.7%) than male youth (25.5%) reported communicating with their parents/guardians about menstruation/wet dreams(p-value<0.005). A statistically significantly higher proportion of male youth (44.9%) than female youth (31.3%) reported communicating with their parents/guardians regarding drug use (p=0.01).

**Table 2:** Percentage of young people reporting communicating with their parents/guardians on various sexual and reproductive health and rights topics

	Average	Food Vendors	House Maids	School Truants	Youth Living with HIV	Youth with Disability	*p*	Male	Female	*p*
Pregnancy & Reproduction	32.8	37.8	17.8	36.5	38.3	20.05	0.03	32.0	33.9	0.72
Sex	32.9	33.8	24.4	33.7	40.8	19.2	0.03	35.5	32.2	0.53
Menstruation wet dreams	40.7	42.0	25.0	37.5	41.9	34.9	0.002	25.5	48.7	<.0005
STDs	38.5	35.7	22.7	41.7	49	26.5	0.00	40.8	37.7	0.57
HIVand AIDS	46.5	36.2	23.8	50.5	66	38.55	<.0005	52.9	43.9	0.11
Family Planning	32.0	40	27.9	30	27.9	19.45	0.04	31.4	32.9	0.77
Homosexuality	20.1	15.7	17.8	17.3	10.4	22.65	<.0005	23.1	18.9	0.37
Drug use	35.8	31.4	20.5	38.9	46.9	24.8	0.01	44.9	31.3	0.01

To assess their attitudes towards communication about SRHR with their parents/guardians, respondents were provided with a list of 16 statements and they were asked to indicate the extent to which they agreed or disagreed with the statements. The results are summarized in Table 3. On average, 52% of the respondents expressed positive attitudes towards communication about SRHR with their parents/guardians.

**Table 3:** Percentage of respondents agreeing with statements on sexual health communication with their parents/guardians

	Average	Food Vendors	House Maids	School Truants	Youth living with HIV	Youth with Disability	Average/Statement
I wouldn't be embarrassed talking to my parents/guardians about sex	53.3	47.8	77.8	55.7	40.8	51.4	54.7
My parents/guardians would love to answer questions about sex	46.0	48.6	51.1	46.7	47.0	45.5	47.8
Parents wouldn't warn and preach to me about sex	40.8	43.4	62.3	31.5	43.7	39.4	44.1
I need to talk to my parents/guardians about sex	55.4	52.2	48.8	64.4	73.4	46.1	57.0
My parents/guardians have enough knowledge about sex	49.7	50.0	45.4	51.5	64.6	47.2	51.7
My parents/guardians would be open to me if we were to talk about sex	43.8	47.8	48.9	45.2	42.8	40.0	44.9
My parents/guardians can still talk to me about sex despite their old age	53.1	52.1	65.9	57.4	55.1	45.4	55.2
My parents/guardians wouldn't be shocked if I tried to talk to them about sex	52.7	54.7	69.0	52.4	51.0	51.4	55.7
It is not difficult getting convenient time to talk about sex with my parents/guardians	57.1	63.3	57.1	57.8	57.1	58.6	58.8
My parents/guardians are not too busy not to get time to talk to me about sex	63.5	58.9	59.5	69.6	70.2	48.6	61.4
My parents/guardians wouldn't ask too many questions if I tried to talk to them about sex	39.6	36.2	60.9	40.2	35.5	38.0	42.2
My parents/guardians would like to hear from me about sex	62.1	68.1	56.1	66.7	67.4	60.9	63.8
We wouldn't be argumentative if we tried to talk about sex with my parents/guardians	59.5	60.9	53.6	70.4	71.4	47.1	60.7
My parents/guardians wouldn't be embarrassed talking to me about sex	55.6	57.4	52.4	60.2	62.5	50.8	56.7
It wouldn't be difficult for me to be open to my parents/guardians in talking about sex	47.1	51.4	40.5	53.4	57.2	39.4	48.4
My parents/guardians would be angry is I tried to talk to them about sex	52.3	55.1	58.1	56.6	57.2	44.4	54.3
Average	52.0	53.0	56.7	55.0	56.1	47.1	53.6

#### Barriers to communication about SRHR between parents/guardians and young people

Several barriers emerged from the qualitative data can be categorized into two major groups. These are cultural barriers and parents’ lack of knowledge/skills about sexual and reproductive health matters. During interviews, respondents explained that it was not in keeping with African culture for parents and their children to discuss issues related to sexuality. For example, respondents observed that:

*“Let’s be honest, can you seriously tell your child, your own son, especially your daughter, my daughter make sure he wears a condom, really? Not in our context “*(male parent, Iringa).

Another respondent added that:

*“These issues should be taught in schools. The most I can do is to warn them, don’t do this and that, but not really sit with them to discuss, to exchange ideas as you put it, there is nothing like that in our culture”* (Female parent, Ilala).

Respondents complained that they lacked basic knowledge and skills about SRH, which limits their confidence to engage their children/youngsters in discussions about sexuality matters. They preferred sex education to be provided in schools rather than at home. Others suggested that there was need for parents’ education so as to equip them with necessary knowledge and skills related to sexuality matters. Some of the respondents’ commented that:

*“You may have the intention to talk to them about these things [SRHR] but some of us have limited partial knowledge about these issues. Maybe we should be taught ourselves first before thinking of teaching them”* (male parent, Iringa).

*“Who should teach the other? These kids nowadays know everything, and we basically know nothing”* (Male parent, Ilala).

## Discussion

Using a cross-sectional survey design and questionnaires as the principal data collection tools, this study assessed marginalized young people’s access to information and education about sexual and reproductive health. Most adolescents do not have access to comprehensive, quality SRHR education [16, 17]. Many countries do have programs in place to address SRHR education in their policies. However, it was found that most countries do not implement this widely or adhere to international standards [18, 19]. Tanzania has been at the forefront in addressing the sexual and reproductive health needs for young people. Young people’s sexual and reproductive health needs have been mainstreamed in different health policy frameworks, including the national policy on HIV and AIDS Policy and the National Multisectoral Strategic Framework (NMSF) on HIV and AIDS 2008-2012 [20, 21]. The Government of the United Republic of Tanzania has also developed a national Adolescent Sexual and Reproductive Health Strategy (ASRH), which provides guidance on addressing the specific needs of young people’s sexual health as comprehensive sexuality education is not a core part of the school curriculum [22]. Marginalized youth still however, find it difficult to access SRHR education as evident from the results of this study.

The results from this study showed that the level of awareness about HIV and AIDS was almost universal, and that majority of young people perceived the importance of SRHR information highly. This high level awareness of HIV and AIDS among the marginalized young people in Tanzania has been demonstrated by another study which found that the level of awareness among young people with disabilities was almost universal [23]. Nevertheless, though the level of comprehensive knowledge about HIV and AIDS was higher for the youth participating in this study than the national average, only two thirds young people demonstrated comprehensive knowledge about HIV and AIDS, compared to the national average of 46 percent of young people [6]. The higher level of comprehensive knowledge about HIV and AIDS among young people in the study sites maybe attributable to the ongoing TUITETEE project interventions among other factors.

The results of this study also showed that young people depended on multiple sources of information about sexual and reproductive health. The leading sources of SRHR information for the majority of the surveyed young people in this study was peers followed by parents, teachers, radio and television. The importance of different sources varied with respect to each of the youth groups. This implies that that no one source can be said to be absolutely more effective than the other. Nevertheless, evidence shows that some sources are credible and therefore more effective than others in reaching young people, especially those with special needs, as is the case with the TUITETEE project clientele. For example, a review done by the World Health Organization in 2009 showed that effective approaches for reaching young people are those that involved community and religious leaders in promoting youth health and promoting Multisectoral linkages to education and livelihood programs [24]. Other studies have shown that peer education programs are most effective in improving knowledge and promoting attitudinal and behavior change among young people in school and community settings [25].

Parents and teachers alongside peer educators seemed to be the most preferred sources of SRHR information for the majority of young people. However, majority of parents expressed feelings of incompetence in providing SRH information to their youngsters. This trend has been evidenced from other studies conducted on this issue [25]. This suggests the need for providing some basic training programs targeting parents on issues related to sexual and reproductive health education.

It is also important to understand the social and economical barriers that exist to access of information and services to SRHR by the youth. A survey of barriers to sexual reproductive health services and rights among young people in Mtwara District revealed, among other things, that the majority of health service providers were not properly equipped to handle the SRH needs of young people and that the health facilities were generally unfriendly to young people [26]. Studies show that young people mostly value and are attracted to the friendliness of health facilities with respect to confidentiality, short waiting time, low cost and staff friendliness [27, 28]. Lack of education and out-of-school setting also seems to be a main hindrance for the participants surveyed in accessing SRHR services. Despite evidence showing benefits to health when the youth stay in school, 89 million of them were found to be out-of school in SSA [29]. When a girl stays on through secondary school, her chances of contracting HIV infections and of risky sexual activities is reduced significantly [30]. Those out of school were from the poorest regions of their countries and hence were most vulnerable to sexual and reproductive health. Within the school context as well severe shortcomings have been studied while imparting SRHR services. A recent study found that even within the school setting, in many countries, HIV-based skills education imparted varied vastly or was inadequate [31]. This suggests that school and community based peer education programs should be linked with improving the health delivery systems to make them relevant and attractive to young people.

In this study, the youth group with the least access to SRHR information and services were found to be housemaids. This is perhaps because most housemaids are expected to spend most of the time indoors due to the nature of household chores they undertake. There is a need for diversified approach for reaching different groups of young people depending on their needs and circumstances. It is clear that one approach may not be appropriate for groups of young people.

## Conclusion

The marginalized young people’s access to SRHR information and education through different means of communication including parents and teachers as the most preferred ones. However, teachers and parents reported to have very limited knowledge on the subject matter. In light of the findings and conclusions of this study it is clear that multiple approaches are needed to reach and meet the diverse needs of the different groups of young people. In this regard, the following measures are recommended: firstly, strengthening of school, community and health services linkages in the delivery of sexual and reproductive health education to young people is critical. In particular, projects should focus on promoting school and curriculum based delivery of sex education, while actively promoting peer led community based education and improving the youth friendliness of health service delivery points to meet the needs of young people. Secondly, some groups of young people such as housemaids seem to be particularly hard to reach because of their unique circumstances. As such, there is a need to explore further with them and their employers on how best they can be reached for SRH information. It is however acknowledged that identification of housemaids continues to be a limitation. A more systematic evaluation of the TUITETEE project intervention is needed to gather evidence regarding what works and what doesn’t in the context of the project sites.

What is known about this topic

- Majority of young people lack education and access to Sexual and Reproductive Health Rights services;
- Poor access to SRHR is associated with young people’s vulnerability to sexual health risks, such as early pregnancies and sexually transmitted diseases;
- SRHR issues of youth with disabilities are often ignored out in research, programming and policymaking.

What this study adds

- Access to SRH rights information was high among youth with disabilities in Tanzania;
- Housemaids were found to have the least access to SRHR information and services;
- Despite of parents and teachers being most preferred sources of SRHR information for the young people, parents expressed feelings of incompetence in the subject.

## References

[1] UNFPA (2014). UNFPA State of World Population 2014 Report..

[2] UNAIDS (2013). Global Report: UNAIDS Report on the Global AIDS Epidemic..

[3] Braeken D, Rondinelli I (2012). Sexual and reproductive health needs of young people: matching needs with systems. Int J Gynaecol Obstet..

[4] Economic Commission for Africa (2011). African Youth Report 2011: Addressing the youth education and employment nexus in the new global economy..

[5] Singh S, Darroch J, Ashford L (2014). Adding It Up: The Costs and Benefits of Investing in Sexual and Reproductive Health..

[6] National Bureau of Statistics (2012). The 2012 Population and Housing Census (PHC) for the United Republic of Tanzania..

[7] Population Reference Bureau (2015). 2015 World Population Data Sheet..

[8] National Bureau of Statistics (2011). Tanzania Demographic Health Survey 2010..

[9] UNAIDS (2014). Global AIDS response country progress report..

[10] UNICEF (2011). Opportunity in crisis: Preventing HIV from early adolescence to young adulthood..

[11] Alemu T, Fantahun M (2011). Sexual and reproductive health status and related problems of young people with disabilities in selected associations of people with disability, Addis Ababa, Ethiopia. Ethiop Med J..

[12] Maart S, Jelsma J (2010). The sexual behavior of physically disabled adolescents. Disabil Rehabil..

[13] Kissin DM, Zapata L, Yorick R, Vinogradova EN, Volkova GV, Cherkassova E (2007). HIV seroprevalence in street youth, St Petersburg, Russia. AIDS..

[14] Busza JR, Balakireva OM, Teltschik A, Bondar TV, Sereda YV, Meynell C (2011). Street-based adolescents at high risk of HIV in Ukraine. J Epidemiol Community Health..

[15] UNFPA (2010). How universal is access to reproductive health?. A review of the evidence..

[16] United Nations Population Division Assessment of the status of implementation of the Programme of Action of the International Conference on Population and Development: Framework of Actions for the Follow-up to the Programme of Action of the International Conference on Population and Development (ICPD) Beyond 2014..

[17] UNESCO (2014). Comprehensive Sexuality Education: The Challenges and Opportunities of Scaling-up..

[18] UNFPA (2014). Operational Guidance for Comprehensive Sexuality Education (CSE): A focus on human rights and gender..

[19] UNFPA (2014). UNFPA Strategy on Adolescents and Youth: Towards realizing the full potential of adolescents and youth..

[20] (2001). The United Republic of Tanzania prime minister’s office National policy on HIV/AIDS 2001..

[21] The United Republic of Tanzania prime minister’s office The second National Multi-sectoral Strategic Framework on HIV and AIDS 2008-2012..

[22] Mkumbo KA (2009). Content analysis of the status and place of sexuality education in the national school policy and curriculum in Tanzania. Educational Research and Reviews..

[23] Restless Development (2011). Survey on behaviors and attitudes of young people in the Southern Highlands of Tanzania..

[24] WHO (2009). Generating demand and community support for sexual and reproductive health services for young people..

[25] Brieger WR, Delano GE, Lane CG, Oladepo O, Oyediran KA (2001). West African Youth Initiative: outcome of a reproductive health education program. J Adolesc Health..

[26] Mbeba RM, Mkuye MS, Magembe GE, Yotham WL, Mellah AO, Mkuwa SB (2012). Barriers to sexual reproductive health services and rights among young people in Mtwara district, Tanzania: a qualitative study. Pan Afr Med J..

[27] Erulkar AS, Onoka CJ, Phiri A (2005). What is youth-friendly? Adolescents’ preferences for reproductive health services in Kenya and Zimbabwe. Afr J Reprod Health..

[28] Geary RS, Webb EL, Clarke L, Norris SA (2015). Evaluating youth-friendly health services: young people’s perspectives from a simulated client study in urban South Africa. Glob Health Action..

[29] Taylor YS, di Gropello E, Gresham J, Inoue K (2015). Out-of-School Youth in Sub-Saharan Africa?: a policy perspective. World Bank..

[30] Greene ME (2014). Ending Child Marriage in a Generation: what research will it take?..

[31] Ragnar A, Panchaud C, Singh S, Watson K (2014). Demystifying data: a guide to using evidence to improve young people’s sexual health and rights..

